# How many Slovenian family practice attendees are victims of intimate partner violence? A re-evaluation cross-sectional study report

**DOI:** 10.1186/1471-2458-13-703

**Published:** 2013-08-01

**Authors:** Polona Selic, Igor Svab, Nena Kopcavar Gucek

**Affiliations:** 1 Department of Family Medicine, Faculty of Medicine, University of Ljubljana, Poljanski nasip 58, Ljubljana, Slovenia

**Keywords:** Intimate partner violence, Violence prevalence, Physical violence, Psychological violence, Primary care, Risk factors

## Abstract

**Background:**

Intimate partner violence (IPV) can be considered a leading public health problem affecting approximately 50% of women during the course of their lifetimes. This study was carried out with the aim of re-testing the prevalence data and providing sufficient grounds for decision-makers in family medicine in Slovenia to adopt much-needed protocols for IPV management in the field.

**Methods:**

In January 2012, every tenth general practitioner (GP) registered in Slovenia, of a total of 958, was invited to participate in a multi-centre cross-sectional study, and 9.4% of them, working in 90 family practices, agreed to participate. From February 1 to March 1, 2012, they asked every fifth family practice attendee aged 18 years and above, regardless of gender, to participate in the study. The short version of Domestic Violence Exposure Questionnaire was administered to 2572 patients.

**Results:**

In the sample, there were more women (62.9% (n = 1617)). The average age of all the participants was 49.0 ± 16.1 years. Of 2572 participants (95.3% response rate), 17.1% people had been exposed to either emotional or both physical and emotional abuse. The prevalence of psychological violence was 10.3%, and that of concurrent physical and psychological abuse 6.8%, with all the patients exposed to physical IPV disclosing concurrent psychological violence. Female gender and previous formal divorce were risk factors identified in all three multivariate logistic regression models. The odds of concurrent physical and psychological and either type of IPV exposure in patients were lessened by an age of 65 years or above. The odds for either type of IPV were also lower in single people, while in concurrent physical and psychological IPV exposure, living in urban settings acted as a protective factor.

**Conclusions:**

In Slovenian family practice attendees, an IPV exposure prevalence of approximately 17% should be considered a valid estimation.

## Background

Any behaviour which causes physical, psychological or sexual harm by a man or woman’s partner or ex-partner, within an intimate relationship, is usually described as intimate partner violence (IPV). This common problem is a pattern of coercive behaviours that may include repeated beating and injury, psychological abuse with coercive control, sexual assault, progressive social isolation, deprivation, and intimidation [1]. IPV, also known as domestic violence (DV), can be considered a leading public health problem affecting approximately 50% of women during the course of their lifetimes [2].

Estimates of the prevalence of experiences of physical or sexual partner violence in women across their lifetimes range from 15%–71%, with past year estimates ranging between 4% and 54%. This shows women are at a far greater risk of physical or sexual violence from a partner than from anyone else [3]. In Canada, almost one in ten women had previously been threatened or injured by their partners; physical DV had been experienced by 40% of women, while the past-year prevalence was 26%, and as many as half of Canadian women reported some form of physical or mental abuse over the course of their lifetimes [4,5]. A study in the United States (US) found that at least one in every 20 women had experienced domestic violence in the previous year; one in five had experienced violence in their adult lives; and one in three had experienced violence either as a child or as an adult [6]. Undoubtedly, IPV has been shown to be the most common type of violence against women [3], affecting an estimated one in four women across their lifespan [7]. A considerable overlap has been found between physical, emotional and sexual violence, with the estimated lifetime prevalence of physical and/or sexual violence being higher in societies where the use of violence in many situations is a socially-accepted norm [8,9]. Aside from its serious health consequences for women and children, a significant impact of IPV on society, including high financial costs, has been shown [10,11].

In Slovenia, prior to 2008 and the adoption of the Law on the Prevention of Domestic Violence, the only official data on DV was when reports of events meeting the criteria of felony or a criminal act were collected by the police [12]. According to recent police records, most victims of DV in Slovenia are women [13]. In 2011, the number was 1,584 and in 2010 1,909, in a country with only about two million inhabitants; however, in police statistics, a victim of violence is counted only once in the reference year, irrespective of how many times violence was reported, which could distort the figures. In both 2010 and in 2011, ten women were murdered by their intimate partners [13].

The first study on the prevalence of DV reported in primary care was carried out in 2006. It found that 12.8% of family care attendees admitted that they had experienced both physical and psychological violence; 5.9% reported that they had been victims of physical violence and 10.9% said that they had been victims of psychological violence, while 70.4% did not report any form of DV [14]. In another survey in 2007, the prevalence of IPV, the perpetrators, and the readiness of DV victims to seek help was addressed [15]. Of that sample, 12.2% of individuals (7.1% of men and 15.1% of women) reported having been a victim of physical violence in the previous five years; another 29% (15.9% of men and 36.7% of women) had been victims of psychological abuse; and 10.7% of those interviewed had experienced both types of violence (4.1% of men and 14.5% of women), while 69.4% of patients (80.7% of men and 62.7% of women) did not report any kind of IPV-related experience in the previous five years [15]. Another study was performed in 2009, aiming mainly to identify the determinants of exposure to psychological and physical violence in family practice patients, so that GPs would be more able to detect them amongst the large numbers of patients in their practices [12]. Of all the patients, 15.3% reported some type of DV experienced during the previous five years; 5.9% reported physical and 9.4% psychological abuse. Exposure to psychological violence was more prevalent than exposure to physical violence; of women, 20.0% were exposed to either type of violence, compared to 8.0% of male participants. Two risk factors affecting the progression from psychological to physical violence were identified, i.e. the abuse of alcohol in the patient and their unemployment [12]. To test the reliability of the data on the prevalence of IPV in primary care patients, and to determine the associated factors, a systematic cross-sectional survey was performed in 2010 [16]. Of these patients, 17.9% were found to have been exposed to psychological or physical IPV in the past five years. Here the factors that increased the chances of exposure to psychological and physical violence were female gender and formal divorce [16].

Despite the high prevalence of DV, and the proven harmful consequences to health, there is still no consensus on prevention strategies for IPV in family medicine or in Slovenia in general. The present study aimed to re-test the prevalence data and provide sufficient grounds for decision-makers in family medicine to adopt much-needed protocols for IPV management in the field.

## Methods

### Participants: GPs

This cross-sectional study aimed to test the results of a study performed in 2010 [16] which focused on the diversity and geographical representation of family care settings as described by Svab et al. [17]. Within the selected family care settings in 2010, it was mostly those GPs already aware of IPV that participated. The goal of the present study was to avoid a biased approach and include GPs regardless of their concepts and attitudes towards IPV management in family medicine; it was therefore decided to systematically sample the GPs to negate their possible different attitudes toward IPV.

In January 2012, there were 958 family physicians (general practitioners (GPs), i.e. family doctors who have finished four years of specialised training) registered in Slovenia. Every tenth GP listed in the Register of Family Medicine Doctors held at the Medical Chamber of Slovenia was invited to participate in a multi-centre study. After a two week recruitment period, 90 GPs (9.4% of all registered), working in 90 family practices all over the country, agreed to participate and were given written instructions about the approach to the patients, data collection and the provision for possible further help.

### Participants: patients

The participating GPs asked every fifth family practice attendee, regardless of gender, aged 18 years and above, who had visited their GP for health problems, and who were given a physical examination, to participate in the study. After the study aim was explained, the subjects were told that participation was not obligatory. Data collection started on February 1, 2012 and finished either after 30 patients had been interviewed, or on March 1, 2012, whichever was the earliest. Visits for administrative purposes, e.g. chronic patients coming for prescriptions and patients requiring sick leave forms, were excluded. No-one was accompanied by another person. The eligibility criteria were age, purpose of visit (health problems), the absence of dementia or even mild cognitive impairment, and their willingness to participate. The Domestic Violence Exposure Questionnaire used in the 2010 study [16] was administered by the GPs after the examination and consultation about the health problem that was the reason for attendance.

Of 2700 invited patients, 2572 were assessed (95.3% response rate); the 128 (4.7%) people who did not want to participate did not disclose their motivation.

The National Medical Ethics Committee of the Republic of Slovenia approved the protocol of the study.

### Procedure

The eligible patients were asked about the presence of physically violent behaviour perpetrated by their intimate partner (i.e. *In the past five years, have you ever been beaten, slapped, kicked or in any other way exposed to physical violence by your spouse or intimate partner?*). A question about coerced sexual intercourse followed (i.e. *In the last five years, have you been forced into sexual intercourse or any unwanted sexual behaviour?*). However, due to the patients’ negative response to this question (i.e. only ten patients answered “yes”) sexual violence is not presented as a special type of IPV in this study. Psychological violence was screened for by asking *In the past five years, have you been humiliated, subjected to threats, insult or intimidation, or in any way emotionally affected by your intimate partner?*

The interview was ended by the GPs’ invitation to the patients to add or ask anything else. Of all those interviewed, 96 (3.7%) patients asked for a special IPV-related consultation.

### Measures

The short form of A Domestic Violence Exposure Questionnaire, described by Kopcavar-Gucek et al. [16] and developed in previous studies in Slovenian primary care [12,14], was used to test the prevalence of IPV in family medicine attendees. It consisted of questions about gender, age, number of children, marital status, number of divorces, place of residence, and exposure to violence (psychological and physical, including coerced sexual intercourse).

### Data analysis

The sample data were presented as frequencies and percentages. Univariate comparisons were made using the χ^2^ test. Multivariate binary logistic regression analysis was used to determine the risk factors for psychological and physical violence. In multivariate logistic regression modelling, the associations between concurrent physical and psychological IPV, psychological abuse only, and physical and/or psychological IPV, all considered as the dependent variables, and the demographic characteristics of the patients, i.e. the independent variables, were explored. The modelling included all the variables from the questionnaire. With regard to each predictive variable in the logistic model, the Wald χ^2^ value, statistical significance (*P* value), odds ratios (OR), and 95% confidence intervals (CI) were calculated. Results were presented by crude odds ratios, calculated between each variable and the outcome (cOR), and adjusted odds ratios for age, gender and all other variables in the model (aOR) to better reflect possible confounding influences. Statistical analysis was performed with IBM SPSS 20.0 software (IBM Corp., Armonk, NY, USA). *P* < 0.05 was set as the level of statistical significance.

## Results

The prevalence of only psychological violence in the sample was 10.3% (n = 266), and that of concurrent physical and psychological abuse was 6.8% (n = 174). All the patients exposed to physical IPV disclosed concurrent psychological violence. Of the sample, 17.1% (n = 440) people were exposed to either just emotional or both physical and emotional abuse.

### Demographic characteristics of patients

The average age of all participants was 49.0 ± 16.1 (18–93) years; the average age of men was 49.1 ± 15.5 years and of women 48.9 ± 16.5 years (p = 0.719). The average age of patients without IPV experience was 49.3 ± 16.2 (18–93) years; men 49.5 ± 15.3 years and women 49.1 ± 16.7 (p = 0.551), while people exposed to IPV were 47.6 ± 16.0 (18–84) years old, of them men 44.3 ± 16.4 and women 48.3 ± 15.9 years, the latter being almost significantly older (p = 0.056).

More demographic characteristics of the sample are presented in Table 1.

**Table 1:** Sample by demographic characteristics

	**Sample n = 2572 (%)**	**No IPV Exposure n = 2132 (82.9%)**	**Physical and/or Psychological Violence Exposure n = 440 (17.1%)**
**Age (years)**			
up to 35	613 (23.8)	496 (23.3)	117 (26.6)
36-49	755 (29.4)	617 (28.9)	138 (31.4)
50-64	713 (27.7)	598 (28.0)	115 (26.1)
65 and above	491 (19.1)	421 (19.7)	70 (15.9)
**Gender**			
male	955 (37.1)	886 (41.6)	69 (15.7)
female	1617 (62.9)	1246 (58.4)	371 (84.3)
**Intimate partnership status**			
living in intimate partnership	1878 (73.0)	1556 (73.0)	322 (73.2)
ending intimate partnership	372 (14.5)	293 (13.7)	79 (18.0)
single	322 (12.5)	283 (13.3)	39 (8.9)
**Divorce**			
never divorced	2267 (88.1)	1911 (89.6)	356 (80.9)
formally divorced	305 (11.9)	221 (10.4)	84 (19.1)
**Number of children**			
none	570 (22.2)	480 (22.5)	90 (20.5)
one or two	1574 (61.2)	1297 (60.8)	277 (63.0)
three or more	428 (16.6)	355 (16.7)	73 (16.6)
**Residence**			
rural	748 (29.1)	621 (29.1)	127 (28.9)
suburban	429 (16.7)	349 (16.4)	80 (18.2)
urban	1395 (54.2)	1162 (54.5)	233 (53.0)

There were more women exposed to concurrent physical and psychological or just psychological IPV (87.4% and 82.3% respectively; p < 0,001), compared to 58.4% of women who were not exposed to either type of violence. People whose intimate partnership had ended were more likely to be exposed to concurrent physical and psychological IPV (21.8%, p = 0.008), compared to those non-exposed to IPV (13.7%). Concurrent physical and psychological violence and only psychological IPV exposure was associated with divorce (20.7% and 18.0% respectively, p < 0.001), compared to 10.4% of those non-exposed to either type of IPV. The prevalence of concurrent physical and psychological IPV was lower in urban settings (p = 0.006); 54.5% of those non-exposed lived in urban settings compared to 43.1% of exposed. In childless families, there was less emotional abuse (22.5%); of people exposed to psychological IPV, 18.4% of them were childless (p = 0.049).

### Associations between concurrent physical and psychological intimate partner violence exposure and the demographic characteristics of patients: logistic regression modelling

In the regression modelling process, the associations between concurrent physical and psychological IPV and the demographic characteristics of patients were explored. Female gender (aOR 4.64, 95% CI 2.93-7.35, p < 0.001) and previous formal divorce (aOR 2.16, 95% CI 1.40-3.34, p = 0.001) were identified as risk factors, while an age of 65 years or above (aOR 0.46, 95% CI 0.25-0.083, p = 0.009) and living in urban settings (aOR 0.66, 95% CI 0.45-0.95, p = 0.027) lessened the odds of concurrent physical and psychological IPV in patients, explaining 11% of the variance (Nagelkerke R^2^ = 0.109, p < 0.001). More results are presented in Table 2.

**Table 2:** Associations between concurrent physical and psychological intimate partner violence exposure and the demographic characteristics of patients: logistic regression modelling

	**No IPV n = 2132 (%)**	**Physical and Psych. IPV n = 174 (%)**	**cOR (95% CI)**	**p**	**aOR (95% CI)**	**p**
**Age (years)**						
up to 35	496 (23.3)	48 (23.6)	1.00 (reference)		1.00 (reference)	
36-49	617 (28.9)	58 (33.3)	0.97 (0.65–1.45)	0.971	0.91 (0.57–1.43)	0.657
50-64	598 (28.0)	43 (24.7)	0.74 (0.48–1.14)	0.174	0.68 (0.41–1.12)	0.128
65 and above	421 (19.7)	25 (14.4)	0.61 (0.37–1.01)	0.056	0.46 (0.25–0.83)	0.009
**Gender**						
male	886 (41.6)	22 (12.6)	1.00 (reference)		1.00 (reference)	
female	1246 (58.4)	152 (87.4)	4.91 (3.12–7.75)	<0.001	4.64 (2.93–7.35)	<0.001
**Intimate partnership status**						
living in intimate partnership	1556 (73.0)	120 (69.0)	1.00 (reference)		1.00 (reference)	
ending intimate partnership	293 (13.7)	38 (21.8)	1.68 (1.14–2.47)	0.008	1.49 (0.95–2.32)	0.082
single	283 (13.3)	16 (9.2)	0.73 (0.43–1.25)	0.257	0.55 (0.29–1.04)	0.066
**Divorce**						
never divorced	1911 (89.6)	138 (79.3)	1.00 (reference)		1.00 (reference)	
formally divorced	221 (10.4)	36 (20.7)	2.26 (1.52–3.34)	<0.001	2.16 (1.40–3.34)	0.001
**Number of children**						
none	480 (22.5)	41 (23.6)	1.00 (reference)		1.00 (reference)	
one or two	1297 (60.8)	95 (54.6)	0.86 (0.59–1.26)	0.429	0.62 (0.38–1.01)	0.056
three or more	355 (16.7)	38 (21.8)	1.25 (0.79–1.99)	0.339	0.95 (0.53–1.71)	0.864
**Residence**						
rural	621 (29.1)	57 (32.8)	1.00 (reference)		1.00 (reference)	
suburban	349 (16.4)	42 (24.1)	1.31 (0.86–2.00)	0.206	1.20 (0.79–1.89)	0.368
urban	1162 (54.5)	75 (43.1)	0.70 (0.49–1.01)	0.054	0.66 (0.45–0.95)	0.027

*cOR* crude odds ratio.

*aOR* adjusted odds ratio.

*Psych.* psychological.

(Nagelkerke R^2^ = 0.109, χ^2^ = 106.974, df = 11, p < 0.001; logistic regression model adjusted for age, gender, and all other independent variables in the table).

### Associations between psychological intimate partner violence exposure and the demographic characteristics of patients: logistic regression modelling

Table 3 presents a logistic regression model of psychological IPV exposure and its associations. Female gender (aOR 3.25, 95% CI 2.34-4.52, p < 0.001) and formal divorce in the past (aOR 1.82, 95% CI 1.26-2.64, p = 0.001) increased the odds of exposure to psychological IPV in patients, with regression modelling explaining 7% of the variance (Nagelkerke R^2^ = 0.070, p < 0.001).

**Table 3:** Associations between psychological intimate partner violence exposure and the demographic characteristics of patients: logistic regression modelling

	**No IPV n = 2132 (%)**	**Psychol. IPV n = 266 (%)**	**cOR (95% CI)**	**p**	**aOR (95% CI)**	**p**
**Age (years)**						
up to 35	496 (23.3)	69 (25.9)	1.00 (reference)		1.00 (reference)	
36-49	617 (28.9)	80 (30.1)	0.93 (0.66–1.31)	0.688	0.79 (0.54–1.16)	0.234
50-64	598 (28.0)	72 (27.1)	0.87 (0.61–1.23)	0.420	0.75 (0.51–1.12)	0.164
65 and above	421 (19.7)	45 (16.9)	0.77 (0.52–1.14)	0.194	0.67 (0.42–1.05)	0.083
**Gender**						
male	886 (41.6)	47 (17.7)	1.00 (reference)		1.00 (reference)	
female	1246 (58.4)	219 (82.3)	3.31 (2.39–4.59)	<0.001	3.25 (2.34–4.52)	<0.001
**Intimate partnership status**						
living in intimate partnership	1556 (73.0)	202 (75.9)	1.00 (reference)		1.00 (reference)	
ending intimate partnership	293 (13.7)	41 (15.4)	1.08 (0.75–1.54)	0.681	0.88 (0.59–1.32)	0.540
single	283 (13.3)	23 (8.6)	0.63 (0.40–0.98)	0.041	0.63 (0.36–1.08)	0.093
**Divorce**						
never divorced	1911 (89.6)	218 (82.0)	1.00 (reference)		1.00 (reference)	
formally divorced	221 (10.4)	48 (18.0)	1.90 (1.35–2.68)	<0.001	1.82 (1.26–2.64)	0.001
**Number of children**						
none	480 (22.5)	49 (18.4)	1.00 (reference)		1.00 (reference)	
one or two	1297 (60.8)	182 (68.4)	1.38 (0.99–1.92)	0.061	1.19 (0.77–1.84)	0.433
three or more	355 (16.7)	35 (13.2)	0.97 (0.61–1.52)	0.881	0.90 (0.52–1.57)	0.711
**Residence**						
rural	621 (29.1)	70 (26.3)	1.00 (reference)		1.00 (reference)	
suburban	349 (16.4)	38 (14.3)	0.97 (0.64–1.46)	0.870	0.90 (0.59–1.38)	0.642
urban	1162 (54.5)	158 (59.4)	1.21 (0.90–1.63)	0.217	1.11 (0.82–1.52)	0.491

*cOR* crude odds ratio.

*aOR* adjusted odds ratio.

Psychol IPV: exposure to psychological intimate partner violence.

(Nagelkerke R^2^ = 0.070, χ^2^ = 85.397, df = 11, p < 0.001; logistic regression model adjusted for age, gender, and all other independent variables in the table).

### Associations between intimate partner violence exposure (physical and/or psychological) and demographic characteristics of patients: logistic regression modelling

There were higher odds of physical and/or psychological IPV exposure in women (aOR 3.71, 95% CI 2.82-4.88, p < 0.001) and divorcees (aOR 1.97, 95% CI 1.46-2.67, p < 0.001). The odds were lower in single people (aOR 0.59, 95% CI 0.38-0.91, p = 0.017) and those aged 65 years or above (aOR 0.58, 95% CI 0.40-0.85, p = 0.005), with the regression modelling explaining 10% of the variance (Nagelkerke R^2^ = 0.096, p < 0.001). This is presented in Table 4.

**Table 4:** Associations between intimate partner violence exposure and the demographic characteristics of patients: logistic regression modelling

	**No IPV n = 2132 (%)**	**IPV exposure n = 440 (%)**	**cOR (95% CI)**	**p**	**aOR (95% CI)**	**p**
**Age (years)**						
up to 35	496 (23.3)	117 (26.6)	1.00 (reference)		1.00 (reference)	
36-49	617 (28.9)	138 (31.4)	0.95 (0.72–1.25)	0.703	0.83 (0.61–1.13)	0.238
50-64	598 (28.0)	115 (26.1)	0.82 (0.61–1.08)	0.158	0.73 (0.52–1.01)	0.054
65 and abov	421 (19.7)	70 (15.9)	0.71 (0.51–0.97)	0.034	0.58 (0.40–0.85)	0.005
**Gender**						
male	886 (41.6)	69 (15.7)	1.00 (reference)		1.00 (reference)	
female	1246 (58.4)	371 (84.3)	3.82 (2.92–5.01)	<0.001	3.71 (2.82–4.88)	<0.001
**Intimate partnership status**						
living in intimate partnership	1556 (73.0)	322 (73.2)	1.00 (reference)		1.00 (reference)	
ending intimate partnership	293 (13.7)	79 (18.0)	1.30 (0.99–1.72)	0.060	1.08 (0.79–1.47)	0.648
single	283 (13.3)	39 (8.9)	0.67 (0.47–0.95)	0.025	0.59 (0.38–0.91)	0.017
**Divorce**						
never divorced	1911 (89.6)	356 (80.9)	1.00 (reference)		1.00 (reference)	
formally divorced	221 (10.4)	84 (19.1)	2.04 (1.55–2.69)	<0.001	1.97 (1.46–2.67)	<0.001
**Number of children**						
none	480 (22.5)	90 (20.5)	1.00 (reference)		1.00 (reference)	
one or two	1297 (60.8)	277 (63.0)	1.14 (0.88–1.48)	0.326	0.90 (0.64–1.27)	0.552
three or more	355 (16.7)	73 (16.6)	1.10 (0.78–1.54)	0.592	0.95 (0.60–1.40)	0.694
**Residence**						
rural	621 (29.1)	127 (28.9)	1.00 (reference)		1.00 (reference)	
suburban	349 (16.4)	80 (18.2)	1.12 (0.82–1.53)	0.469	1.05 (0.76–1.45)	0.764
urban	1162 (54.5)	233 (53.0)	0.98 (0.77–1.24)	0.871	0.92 (0.72–1.18)	0.512

*cOR* crude odds ratio.

*aOR* adjusted odds ratio.

(Nagelkerke R^2^ = 0.096, χ^2^ = 152.081, df = 11, p < 0.001; logistic regression model adjusted for age, gender, and all other independent variables in the table).

**Figure 1:**
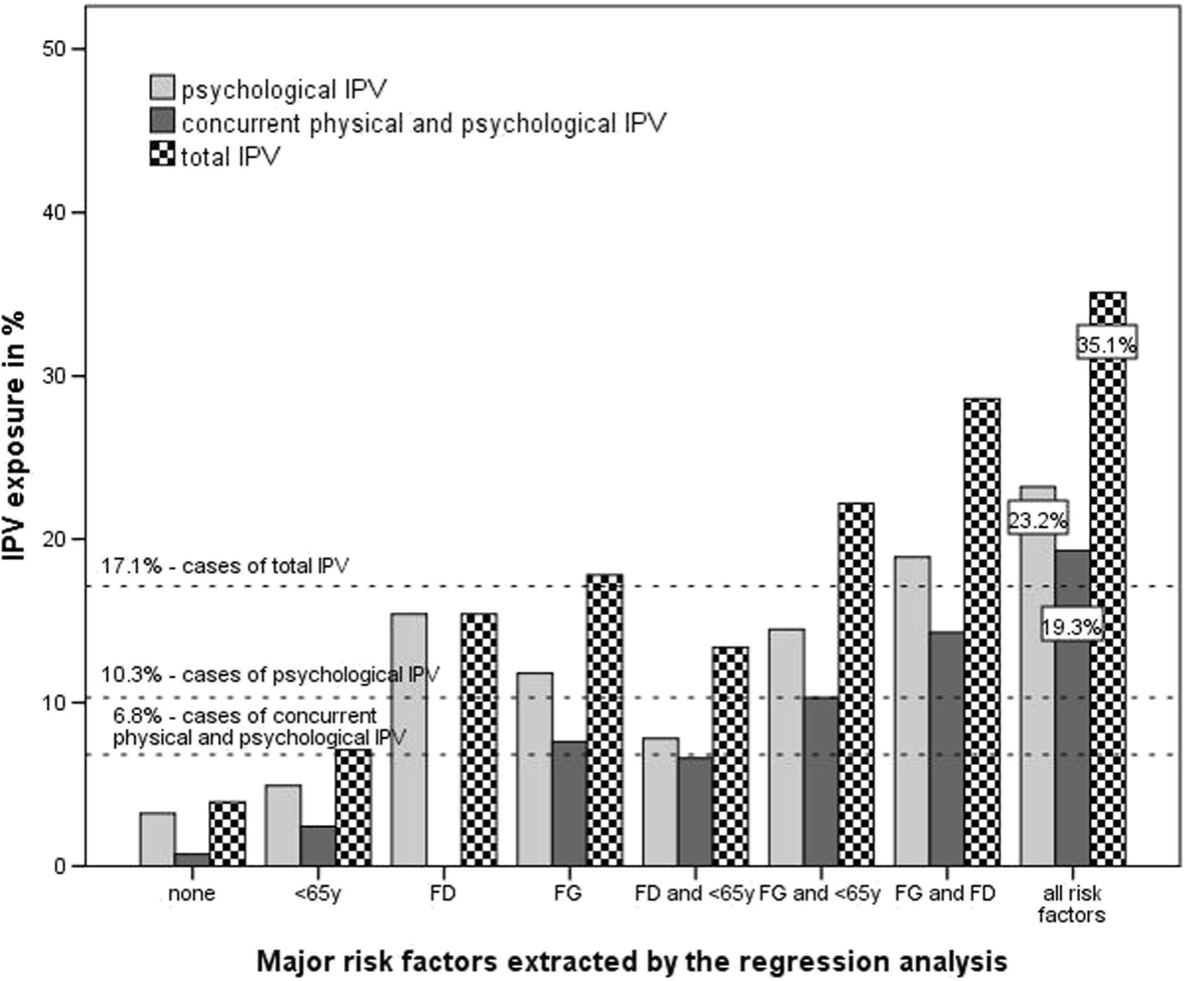
Major risk factors for IPV exposure.

Major risk factors for IPV exposure are presented in Figure 1.

## Discussion

The study confirmed the prevalence of IPV (17.1%) and the female gender’s association with concurrent physical and psychological abuse, with only psychological abuse and with physical and/or psychological IPV exposure (Tables 2, 3 and 4, Figure 1). In comparison to a representative sample of Slovenian family practice attendees [17], in our sample there were more women (62.9% *vs.* 54.8%) and the mean age was slightly younger (49.0 ± 16.1 years *vs.* 51.7 ± 19.0). The predominance of women may have affected the gender distribution of violence in the sample. Aside from female gender, a previous formal divorce was identified as a risk factor of IPV exposure (Tables 2, 3 and 4, Figure 1), although it remains unclear whether the IPV appeared before, during or after the divorce. More in-depth exploration of this factor is needed in further research.

On the other hand, it may be of no surprise that a single marital status acted as a protective factor for either type of IPV (Table 4). However, an age of 65 years and above lessened the odds for either type of IPV (Table 4) and for concurrent physical and psychological abuse (Table 2), bringing into focus the somewhat different aspect of the wellbeing of the elderly. Concordant with the 2010 study [18], the fact that the elderly are less at risk than the younger population may be due to lifestyle changes in the last decades in Slovenia. Aside from that, the increase in life expectancy, the effectiveness of diagnostic and therapeutic interventions, and quality of life, might empower individuals of over 65 to be less vulnerable to DV. Although many studies have demonstrated that the epidemiology and type of abuse in older people may be different in various countries, it has been shown that its prevalence ranges between 3.2 and 27.5% in the general population [18]. Most research has been conducted in Western countries in cognitively intact elderly patients, with reported estimates ranging from 2.2% to 18.4% [19-21]. Another study in Canadian family practices suggested rates of elderly abuse in the range of 12.0% -13.3% [22]; however, the focus of our study was IPV exposure, and abuse of the elderly is a much broader phenomenon. Regardless of that, our results are to be interpreted with great caution and their reliability should be retested on a representative sample of the general population.

Living in urban settings lessened the odds for concurrent physical and psychological IPV (Table 2), which could reflect specific cultural contexts [8,9] and also more opportunities for IPV victims to enter into the social services, mental health, and judicial systems.

A major concern for the provision of services for IPV victims is to ensure that women are not further victimized by the health sector, but are treated sensitively. The 2006 Slovenian study [14] showed that in one fifth of cases the GPs did not do anything when patients asked for help in cases of DV. Physicians suggested secondary care treatment to about a quarter of the victims, and they tried to discuss the problem with two-fifths of those seeking help [14]. Related to this, an important issue of concern in Slovenian family medicine is the challenge of ensuring that health personnel are appropriately trained to provide support services.

In spite of the identified 17.1% IPV prevalence, only 3.7% of all the interviewed patients asked for a special IPV-related consultation. It can be speculated that the patients were afraid that GPs would discount their stories, would be uninterested, would ignore the situation or would focus on physical symptoms. It is also worth mentioning that of all 440 people exposed to either type of IPV, only 10(2.3%) disclosed coerced sexual intercourse. This could have been due to the GP’s inappropriate approach, the patient’s shame, or lack of trust within the doctor-patient relationship. Given that, GPs might often have been missing opportunities to detect victims of abuse in a variety of clinical situations. The detection of domestic violence by GPs might alter both the diagnostic and treatment plans for these patients. However, dealing with patients suspected of being physically abused, sexually abused, or involved in other violent acts was the least common ethical dilemma (<0.1%) among Slovenian GPs [23]. On the other hand, in a study aiming to determine the prevalence of difficulties in managing ethical dilemmas in Slovenian family practice, Klemenc-Ketis et al. [24] found the most difficult ethical issues for GPs were abandoned and unattended patients and patients with insufficient means of support (48.6%), as well as suspicion of physical abuse, sexual abuse, or other criminal behaviour exposure in patients (40.9%).

Therefore it is of the utmost importance to develop and adopt clear procedures and guidelines in Slovenian family medicine for primary care providers, i.e. GPs and nurses, stating their required roles and competencies, and to establish systems for supervision and ongoing monitoring. The policies, protocols and other tools and procedures for IPV responses will help to streamline IPV services as part of the delivery of primary care, and contribute to an improvement in their implementation. As an aspect of this, sustainability of training in the long term is a common challenge for family medicine in Slovenia. Nowadays, lack of record-keeping, time–pressure and various attitudes towards IPV could be constraints on GPs [14], also affecting the results of the present study.

### Limitations

Although this study reduced the bias in GPs’ participation, it also had some limitations. It failed to provide valid data on sexual abuse and did not query specific forms of psychological abuse or neglect. Moreover, each type of abuse was assessed with only one short question, which was due to limited resources of various kinds. The authors are aware that to identify the type and dynamics of abuse adequately, more specific questionnaires with comprehensive, behaviourally defined descriptions of interpersonal violence events in closed questions must be used. Regression modelling explained only 11% of the variance of concurrent physical and psychological IPV, 7% of the variance of psychological IPV exposure and 10% of the variance of either type of IPV, which clearly indicates that modifications in further study design are required. More variables should be analyzed in future research to expand the explained variance, especially to reveal as yet unrecognized determinants of IPV exposure.

Both the previous study and this one show that IPV in Slovenian family medicine attendees is widespread, although the validity of the data may be limited, and more due to psychological factors than to real experience or lack thereof. As emphasized by Feder et al. [25], asking people about a longer period of time or recent experience could both be potentially problematic, i.e. recall bias may be present in responses about a longer period of time, as in the present study, while participants in studies of past year violence might have had insufficient time to acknowledge or identify their abuse experiences as such.

The cross-sectional survey design is inherently limited and, together with reliance on self-reported data, raises questions about the potential for method variance (i.e. same-source measurement bias) to account for our findings. Although the phenomenon being studied could have been assessed only by asking patients to report their experience or perception, it would be useful in further research design for some measures to be incorporated (e.g. medical records to obtain the exact health related data etc.) and measured over time (a prospective study), to mitigate the potential effects of method variance.

## Conclusions

An approximately 17% prevalence of IPV exposure in Slovenian family practice attendees leaves no doubt about the seriousness of the problem. It is therefore of the utmost importance that family medicine professionals receive proper IPV-related education and comprehensive training, to enable them to understand and recognise IPV and its health effects on their patients. Aside from facilitating GPs with training, professional policies are needed. Therefore, in family medicine in Slovenia, it is necessary to introduce and develop IPV-related referral resources, policy guidelines and protocols.

## Competing interests

The authors declare that they have no competing interests.

## Authors’ contributions

PS, IS and NKG conceived the study. PS carried out the coordination and drafted the manuscript. IS participated in interpretation and helped to draft the manuscript. NKG participated in the execution of the study. All the authors read and approved the final manuscript.

## Authors’ information

PS: PhD Clinical Psychology, Senior Researcher and Assistant Professor at the Department of Family Medicine. IS: PhD Family Medicine, Professor, Head of the Department of Family Medicine. NKG: PhD student.

## Pre-publication history

The pre-publication history for this paper can be accessed here:

http://www.biomedcentral.com/1471-2458/13/703/prepub
